# 2-Year survival benefit from immunotherapy for squamous cell cancer with cancer of unknown primary in mediastinum: a case report

**DOI:** 10.3389/fonc.2023.1242460

**Published:** 2023-10-11

**Authors:** Wei Zhao, Nan Zhao, Manze Zhang, Zhihua Li, Ning Wang, Wennan Shen, Yuemei Dong, Yanli Nie, Zhaoxia Li

**Affiliations:** ^1^ Oncology Department, PLA Rocket Force Characteristic Medical Center, Beijing, China; ^2^ Department of Gastrointestinal Medical Oncology, Hubei Cancer Hospital, Tongji Medical College, Huazhong University of Science and Technology, Wuhan, China

**Keywords:** cancer of unknown primary, immunotherapy, mediastinum metastasis, squamous cell cancer, case report

## Abstract

Cancers of unknown primary (CUP) account for 2%–5% of all diagnosed cancers and are always characterized with fast-paced aggression, early metastasis, and unpredictable spread patterns Mediastinum metastasis with unknown primary origin is extremely rare and with a poor prognosis and short survival. There is no literature to refer to for its treatment. Here, we reported a case of squamous cell CUP in the mediastinum. A 50-year-old male patient was admitted after multi-line treatment of low differentiated squamous cell carcinoma in the mediastinum diagnosed 8 months before. In August 2019, the patient went to a local hospital for cough and dyspnea for 2 weeks. Then, he was diagnosed with squamous cell carcinoma of unknown primary origin with multiple lymph nodes metastasis. The patient was featured with programmed cell death-ligand 1 (PD-L1) expression strongly positive in 90% of tumor cells and the combined positive score of 90 and a tumor mutation burden of 1.79 MUts/Mb and microsatellite stable phenotype. The patient was treated with anti-programmed cell death-1 (PD-1) antibodies in combination with chemotherapy and responded to the treatment. The patient showed stable disease to multi-line immunotherapy for more than 7 months and finally got a clinical benefit of 2-year survival benefit. In conclusion, immunotherapy targeting PD-1/PD-L1 in combination with chemotherapy may play a crucial role in the later-line treatment and palliative care of CUP.

## 1 Introduction

Cancers of unknown primary (CUP) are defined as histologically confirmed, metastatic carcinomas or undifferentiated neoplasm, in which the primary tumor site cannot be identified according to a standardized diagnostic evaluation (1–3). CUP, which account for 2%–5% of all diagnosed cancers, are always characterized with fast-paced aggression, early metastasis, and unpredictable spread patterns (3–5). According to autopsy analysis, the most common sites of CUP involve lung, pancreas, hepatobiliary, kidney, bowel, genitourinary, and stomach (6, 7). Adenocarcinoma carcinoma is the most common histology of CUP, while squamous cell carcinoma contributes for approximately 15% cases of CPU (8). Metastatic squamous cell carcinoma with unknown primary has a poor prognosis, has a survival time of 4–12 months, and is generally insensitive to conventional chemotherapy (1). Choosing appropriate treatment regimen for CUPs with optimal clinical benefit has perplexed physicians all the time. In the molecular era, the situation has been improved, since predictive biomarkers has been used to guide targeted therapy, integrating the comprehensive genomic profiling of CUP (9). Several predictive biomarkers, which are suggestive of a likely response to immune checkpoint inhibitors, including microsatellite instability, tumor mutation burden, and high programmed cell death-ligand 1 (PD-L1) expression, may be also appropriate for an appreciable proportion of CUP (3, 10). Furthermore, according to comprehensive analyses of genomic profiling of CUP in two studies, approximately 22% of CUP were PD-L1 positive (10) and 14% CUP cases with high PD-L1 expression with tumor proportion score (TPS) ≥50% (11). It is indicated that PD-L1-positive CUP cases may have a feasibility to respond to immunotherapies targeting programmed cell death protein-1 (PD-1)/PD-L1 axis. Immunotherapy such as immune checkpoint inhibitors (ICIs) has become widely used in the treatment of several malignancies (12, 13). KEYNOTE-024 trial revealed that pembrolizumab was associated with improved overall survival (OS) and progression-free survival (PFS) in NSCLC patients with PD-L1 tumor proportion score (TPS) ≥50% (14). Furthermore, KEYNOTE-048 results showed that pembrolizumab improved OS in patients with R/M HNSCC as PD-L1 increases (15). Thus, CUP patients with PD-L1-positive expression may benefit more from anti-PD-(L)1 treatment. Tislelizumab is an anti-PD-1 monoclonal antibody, which has a significant anti-tumor activity and good safety in various tumors when subjected to monotherapy or combined with chemotherapy (16–19).

Multiple lymph node involvement in the mediastinum with unknown primary origin is extremely rare (20, 21) and always characterized with poor prognosis and short survival. There is no literature to refer to for its treatment. Here, we present a case of squamous cell cancer with unknown primary origin in the mediastinum, who benefited from immunotherapy and chemotherapy.

## 2 Case presentation

A 50-year-old male patient was admitted to the hospital for further treatment after multi-line treatment of low differentiated squamous cell carcinoma in the mediastinum diagnosed 8 months before. The patient was previously in good health without smoking history and no family history of cancer. In August 2019, the patient went to a local hospital for cough and dyspnea for 2 weeks. Computed tomography (CT) screening and positron emission tomography (PET)/CT revealed multiple swollen lymph nodes in the mediastinum. Hypermetabolic foci were not detected in other remaining sites of the body. The patient was diagnosed with squamous cell carcinoma of unknown primary origin with multiple lymph nodes metastasis based on the histopathological examination of biopsy specimens. According to results of analyses of blood and tissue samples by next-generation sequencing, the PD-L1 expression was strongly positive in 90% of tumor cells and PD-L1 combined positive score (CPS) was 90. In addition, the patient was characterized with a tumor mutation burden (TMB) of 1.79 MUts/Mb and a microsatellite stable phenotype.

From 7 September 2019 to 16 November 2019, the patient was treated with four cycles of camrelizumab (200 mg d1, Q3W) plus endostar combined with chemotherapy (vinorelbine plus cisplatin). He was assessed as having a stable disease (SD) at the end of the second cycle and progressive disease (PD) at the end of the fourth cycle on 28 November 2019. The patient had developed facial intermittent bleeding reactive cutaneous capillary endothelial proliferation. Then, he was treated with three cycles of camrelizumab plus endostar combined with paclitaxel (for one cycle and discontinued for allergy to paclitaxel) or carboplatin (for two cycles) from 1 December 2019 to 12 January 2020. Then, treatment discontinued due to coronavirus disease 2019 (COVID-19); thereafter, he was treated with anrotinib (10 mg, D1-14, Q21D) for 2 months and then was assessed as PD in March 2020. In May 2020, with worsened cough and dyspnea, he came to our hospital for further treatment.

**Figure 1:**
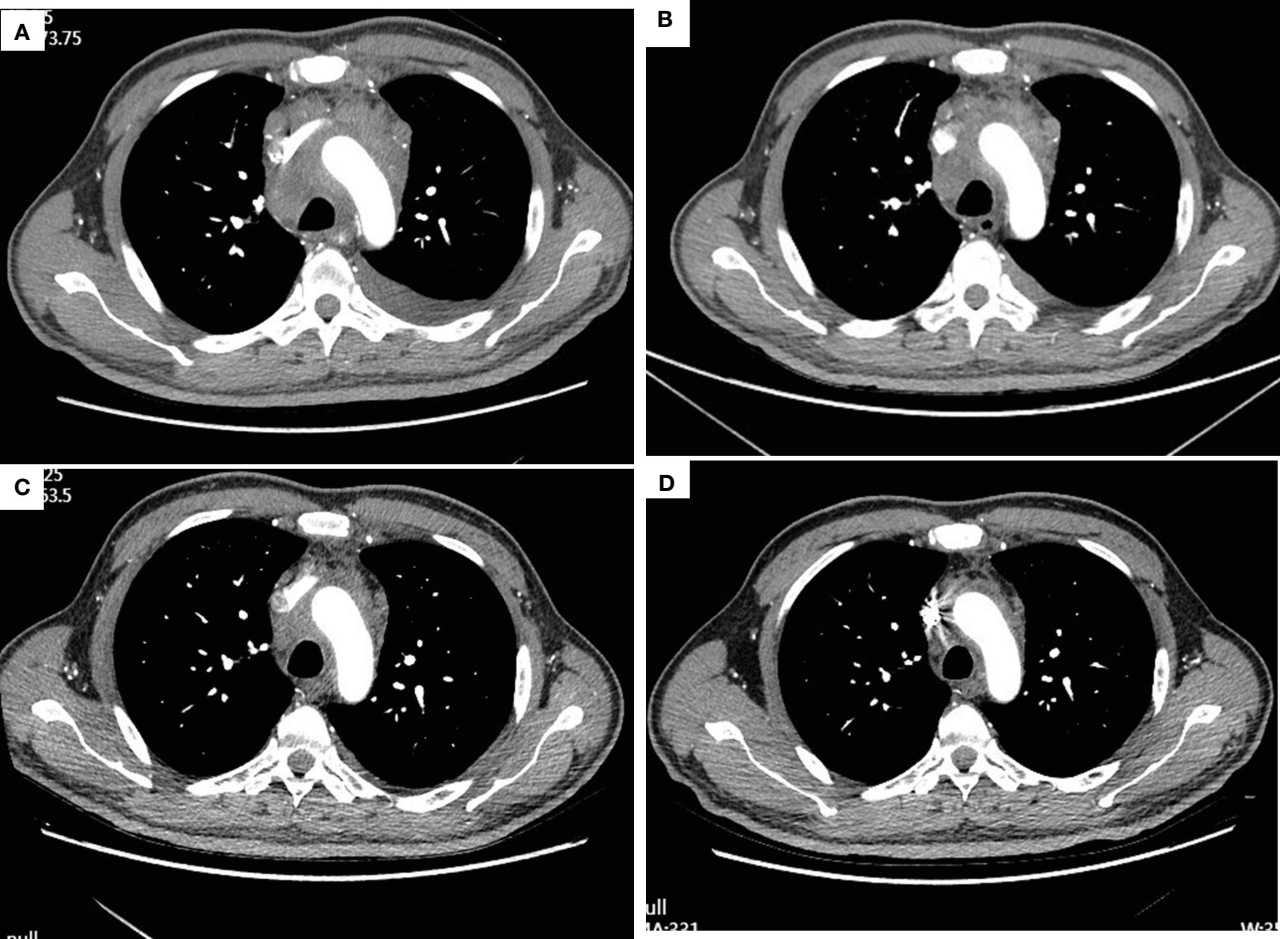
Thorax CT imaging of the patient before and after treatment of tislelizumab plus albumin-bound paclitaxel. **(A–D)** show the CT imaging at baseline, the end of the second cycle, the third cycle, and the fifth cycle, respectively. **(A)** Baseline imaging on 19 May 2020; **(B)** imaging on 16 July 2020, after two cycles treatment; **(C)** imaging on 9 September 2020, after three cycles treatment; **(D)** imaging on 3 November 2020, after five cycles treatment.

After admission, vital signs examination found that the patient had a heart rate of 110/min, breath of 30/min, and a lower breath sound of the left lower lung. Tumor biomarker test showed positive cytokeratin and normal level of carcinoembryonic antigen and squamous cell carcinoma antigen. There was no other abnormality. Thorax CT imaging showed irregular masses involving the ascending aorta, the aortic arch, the superior vena cava, the pulmonary artery, and the trachea and main bronchi; multiple enlarged lymph nodes in the mediastinum; and diffuse irregular thickening of the pericardium (**Figure 1A**). No lesion was found by abdomen CT and head MRI. The pathological consultation suggested a low-differentiated squamous cell carcinoma in the mediastinum. The multidisciplinary team recommended radiotherapy first aiming to relieve the compression symptom of wheezing, cough, and expectoration.

**Figure 2:**
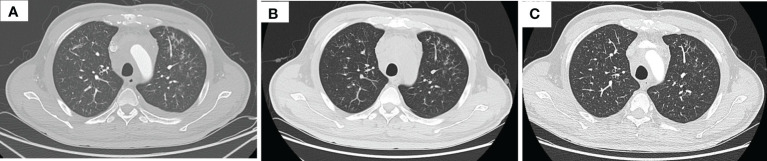
Thorax CT imaging indicates pulmonary inflammation after treatment of tislelizumab plus albumin-bound paclitaxel, which disappears in approximately 2 months when monitored closely. **(A)** Imaging on 16 July 2020, after two cycles immunotherapy, inflammation focally affecting the left lung; **(B)** imaging on 22 July 2020, 1 week after the time of **(A)**, the inflammation is still in the left lung; **(C)** imaging on 9 September 2020, after three cycles immunotherapy, the inflammation basically subsided.

After three times treatment by linear accelerator radiotherapy, the aforementioned symptoms significantly worsened. He got better after drainage of pleural effusion and discontinued radiotherapy. Then, he was treated with tislelizumab (200 mg D1, 21 days a cycle) plus albumin-bound paclitaxel (200 mg D1, 100 mg D8, 21 days a cycle) for five cycles from 11 June 2020 to 10 October 2020. The symptoms improved further since the fifth day after immunochemotherapy. The patient was assessed as having stable disease (SD) at the end of the second cycle treatment (**Figures 1B–D**). The response was assessed as SD at the end of the fifth cycle with the lesions in mediastinum shrinking successively. However, the patient was indicated with pulmonary fibrosis, which might be grade 1 checkpoint inhibitor pneumonitis (CIP) (**Figures 2A, B**), grade 2 leukopenia, and grade 3 thrombocytopenia. The CIP was closely monitored and got better when assessed on 22 July 2020 (**Figures 2B, C**). The hematological toxicities recovered after symptomatic treatment.

Thereafter, the patient was treated with tislelizumab as maintenance therapy for three cycles from 4 November 2020 to 24 December 2020. No tislelizumab-related adverse event occurred. Then, treatment discontinued due to the COVID-19 pandemic. Until 27 January 2021, the patient was confirmed PD with mediastinum space occupying focus and multiple liver metastases, which was considered as new.

**Figure 3:**
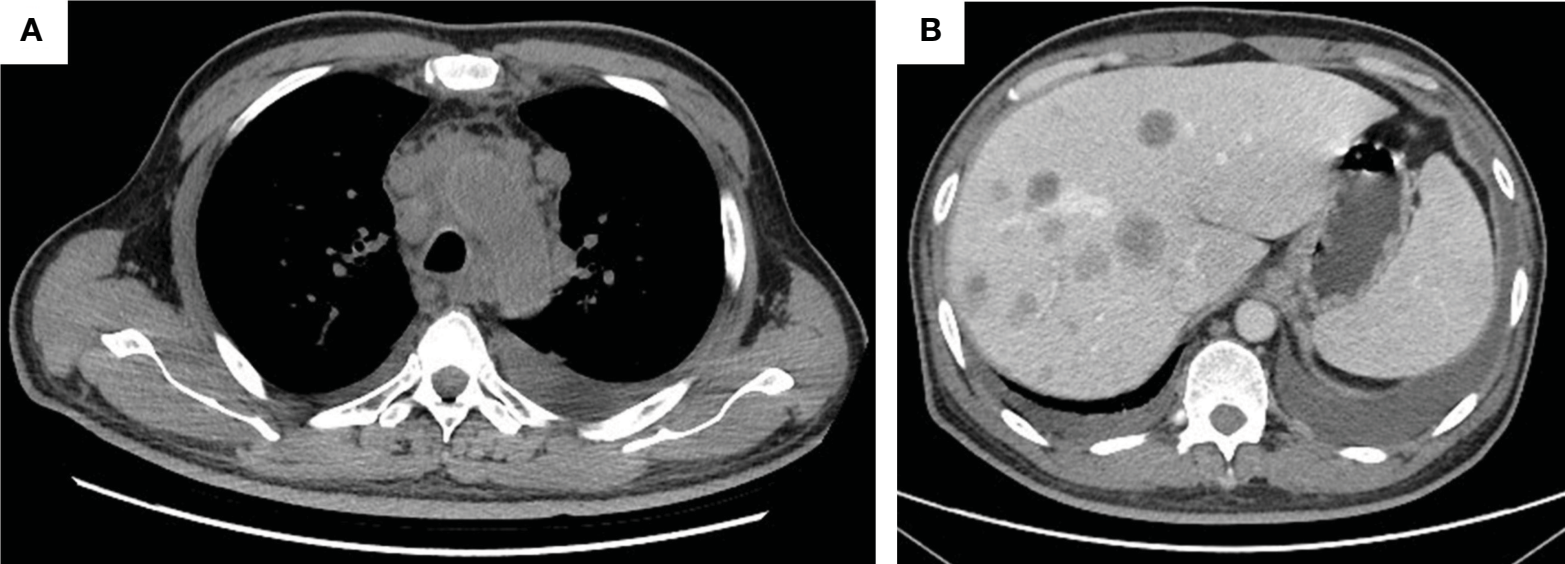
The tumor progression by thorax and abdomen CT imaging. **(A)** Thorax imaging indicates enlarged lesions in the mediastinum on 16 March 2021; **(B)** abdomen imaging on 16 March 2021 indicates multiple live metastases.

**Figure 4:**
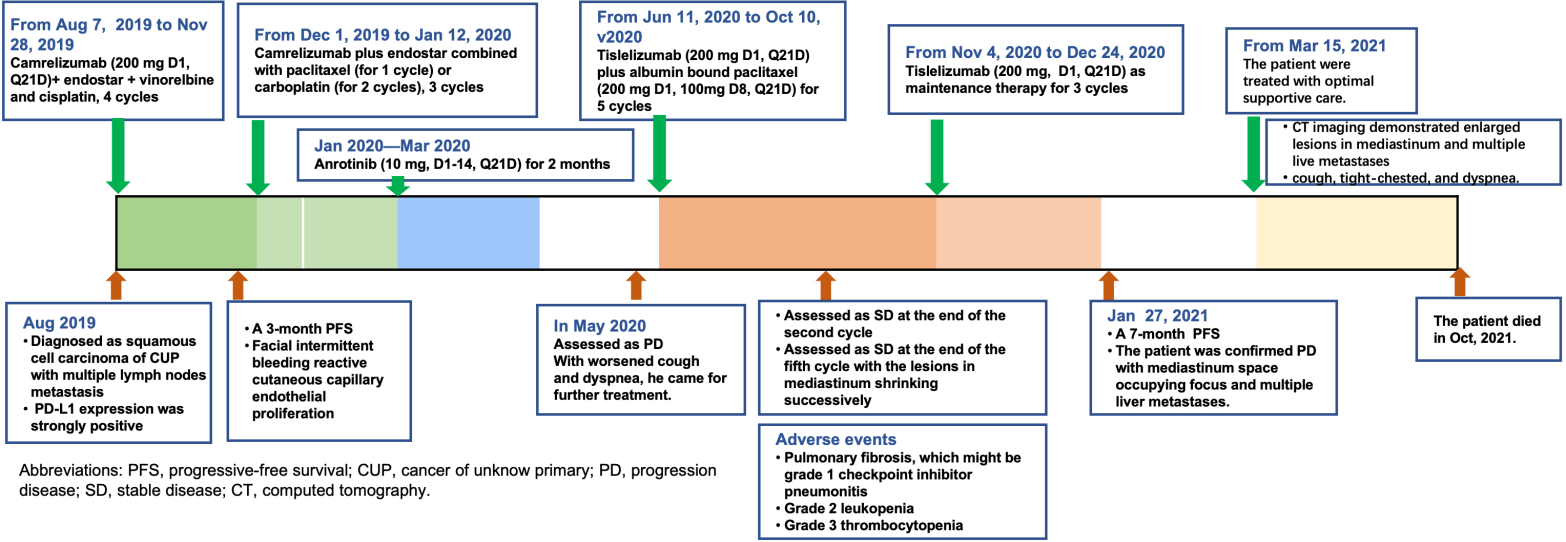
Summary of treatment timeline.

The patient was admitted in our hospital for cough, tight chest, and dyspnea on 15 March 2021. CT imaging demonstrated enlarged lesions in the mediastinum and multiple live metastases (**Figures 3A, B**). In consideration of the poor physical condition, the patient was treated with optimal supportive care. The patient died in October 2021. The treatment timeline is summarized in **Figure 4**. The biochemical analysis results of CEA, neuron-specific enolase (NSE), cytokeratin 19 fragments (CYFRA21-1), and albumin were collected. The change in cytokeratin is summarized in **Supplementary Figure 1**.

## 3 Discussion

In this report, we reported a rare case of squamous cell carcinoma of unknown origin with highly positive PD-L1 expression in the mediastinal metastatic lymph nodes, who benefitted from anti-PD-1 targeted immunotherapy plus chemotherapy and gained a PFS of over 7 months and OS of 26 months. This is the first reported case of squamous cell carcinoma of unknown origin in the mediastinal metastatic lymph nodes, who benefitted from the strategy of immunochemotherapy with prolonged PFS and OS. Biomarker analysis of PD-L1 expression plays a pivotal role in choosing the optimal treatment regimen.

As to the CUP characteristics of aggressive and early metastasis and unpredictable patterns of spread, it is of importance to have the histopathological evaluation of the primary site and identify subtypes of specific tumor, which can be more definitively treated or have superior outcomes (3, 8). However, it is sometimes difficult and time consuming to identify the primary site of tumor, with a median time of 42 days from CUP diagnosis until pharmacotherapy treatment initiation (22). In this case, however, even various modern medical detective methods of imaging, including PET-CT, failed to identify the primary origin of malignancy in this case. In addition, the multiple lymph node involvement in the mediastinum is extremely rare. The patient had been diagnosed as CUP with high expression of PD-L1 and treated with anti-PD-1 immunotherapy when he showed up at our medical center and had a best response of SD to previous treatment. The high expression of PD-L1 served as a predictive biomarker to immune checkpoint blockade. According to a comprehensive analysis of CUP for the biomarkers of response to immune checkpoint blockade therapy, PD-L1 was expressed in 22% of CUP patients (10). It is reasonable to suggest that ICIs could serve as the basal treatment of high PD-L1 expression. However, only a subset of CUP patients may respond to ICIs, considerations integrated with genomic profiling, including TMB and detection of immune or inflammatory biomarkers in the tumors (23).

In this case, tislelizumab plus albumin-bound paclitaxel was chosen as the fourth-line treatment. The patient achieved SD from combined therapy, and a PFS of over 7 months with acceptable adverse event, which was proved to be an effective therapy scheme (24). The OS was over 26 months, which was significantly longer than that reported in the current literature.

In a nutshell, this study reports the first clinical evidence that ICIs plus chemotherapy in a patient with squamous cell cancer with unknown primary origin in the mediastinum, introduced successful whole management more than 26 months of OS benefit. During the course, further assessment of the optimal biomarker should be taken account in selection of checkpoint immunotherapy for CUP.

## Data availability statement

The original contributions presented in the study are included in the article/**Supplementary Material**. Further inquiries can be directed to the corresponding authors.

## Ethics statement

Written informed consent was obtained from the individual’s next of kin for the publication of any potentially identifiable images or data included in this article.

## Author contributions

WZ and ZXL contributed to conception and design of the study and approved the final manuscript. NZ and MZ contributed to collecting and analyzing radiology figures and writing the initial manuscript. WZ and YN were responsible for writing and polishing the manuscript. ZHL, NW, WS, and YD played a role in summarizing the data. All authors contributed to the article and approved the submitted version.

## Supplementary material

Supplementary Figure 1 Change in cytokeratin.
