# Supplementary figures and images for: 2-Year survival benefit from immunotherapy for squamous cell cancer with cancer of unknown primary in mediastinum: a case report
Source: Front Oncol. 2023 Oct 11;13:1242460. doi: 10.3389/fonc.2023.1242460 (PMC10598860; doi:10.3389/fonc.2023.1242460)

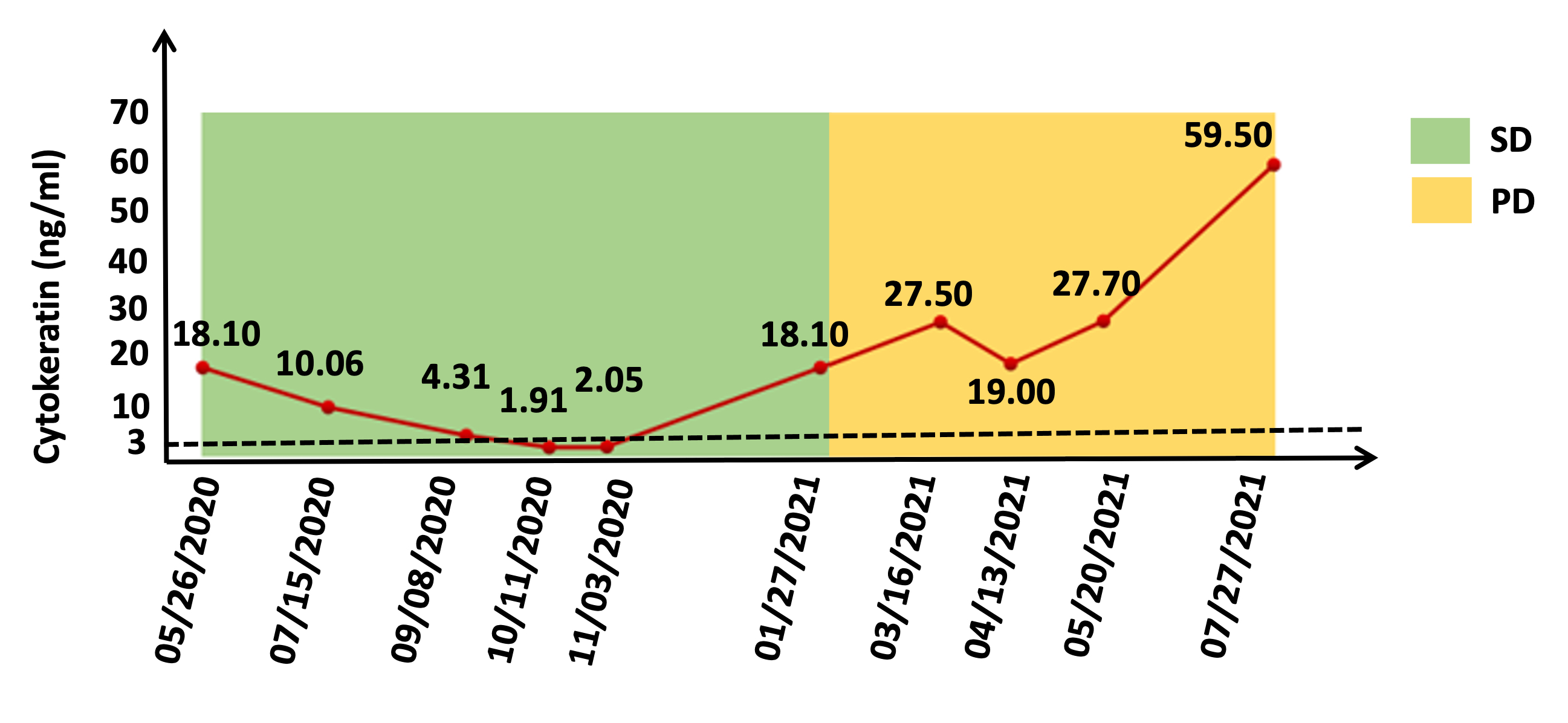

Supplement: Supplementary Figure 1 — Change in cytokeratin. [file Image_1.jpeg]
